# StripePoint-YOLO: Task-Adaptive Detection of Multi-Type Weld Seam Keypoints in Noisy Industrial Welding Scenes

**DOI:** 10.3390/s26154980

**Published:** 2026-08-06

**Authors:** Mingyue Yang, Shizhen Li, Xiaoyan Sun, Hougao Wang, Ang Gao, Fuxin Du, Chao Chen

**Affiliations:** 1 School of Rail Transportation, Shandong Jiaotong University, Jinan 250357, China; 25221004@stu.sdjtu.edu.cn; 2 Shantui Construction Machinery Co., Ltd., Jining 272073, China; lishizhen@shantui.com; 3 MH Robot & Automation Co., Ltd., Weifang 262200, China; sunxiaoyan@mhauto.cn; 4 Shandong Nuclear Power Equipment Manufacturing Co., Ltd., Yantai 265100, China; wangli0212190@163.com; 5 School of Mechanical Engineering, Shandong University, 17923 Jingshi Road, Jinan 250061, China; 202414363@mail.sdu.edu.cn (A.G.); dufuxin@sdu.edu.cn (F.D.); 6 Shandong Province Key Laboratory for Electromagnetic Control and Multifunctional Integration Technology of Aerospace Electromagnctic Functional Structure, Jinan 250061, China

**Keywords:** robotic welding, weld seam keypoint detection, structured-light vision, noisy industrial scenes, physics-driven data augmentation, mean center error

## Abstract

To address the difficulty in stably detecting weld seam keypoints under complex industrial interferences, such as intense arc light, spatter, reflection, and partial occlusion, this paper proposes a lightweight weld seam keypoint detection model named StripePoint-YOLO. The proposed method formulates five typical types of weld seams as a unified detection-based keypoint localization task. Built upon YOLO11n, the model introduces a P2 detection head to enhance shallow high-resolution feature representation for keypoints and adopts SPDConv to reduce the loss of local details caused by early-stage downsampling. Meanwhile, the P5 detection output layer is removed, while its deep semantic features are retained for top-down feature fusion. This design reduces the negative influence of redundant coarse-scale predictions on the center localization of tiny keypoints. For optimization and training, WIoU v3 and NWDLoss are adopted as a joint regression loss to improve the stability of small-scale keypoint bounding box regression. In addition, an online physics-driven data augmentation strategy, OPDDA, is designed to simulate welding disturbances such as arc light, spatter, and dynamic occlusion. Experimental results show that StripePoint-YOLO achieves an mAP@50-95 of 80.46%, a Mean Center Error (MCE) of only 2.33 px, a parameter count of 1.86 M, and a computational cost of 19.42 GFLOPs, while reaching an inference speed of 159.80 FPS under the reported hardware configuration. Further MCE visualization and localization error analysis demonstrate that the proposed method maintains stable keypoint center localization across multiple weld seam types and complex noisy scenarios, verifying the effectiveness of StripePoint-YOLO for accurate and efficient weld seam keypoint detection in industrial welding images.

## 1. Introduction

Robotic welding can reduce the dependence on manual welding experience while improving production consistency and level of automation in complex manufacturing environments. However, due to workpiece assembly errors, thermal deformation, and variations in groove geometry, offline-taught trajectories often deviate during actual welding. As a result, welding robots must rely on visual perception for weld seam recognition, geometric measurement, and trajectory correction [1,2]. Active vision sensing methods usually project structured light or laser stripes onto the welding area and use cameras to capture geometric information about the weld seam. Related reviews have categorized them into key application directions such as weld seam tracking, weld bead defect detection, three-dimensional weld pool measurement, and welding path planning [3,4]. In practical welding processes, arc light, spatter, fumes, and surface reflections can degrade the imaging quality of laser stripes and reduce the stability of weld feature point extraction.

Traditional laser vision methods usually obtain weld seam feature points through threshold segmentation, centerline extraction, morphological processing, edge detection, or curve fitting. These methods offer good interpretability and real-time performance when the laser stripes are clear and the noise level is low. For example, Xiao et al. [5] proposed an improved Snake model to enhance the stability of feature extraction for multi-pass weld tracking in multi-layer and multi-pass welding under irregular stripe patterns and welding noise. However, such methods generally rely on stripe continuity, manually tuned image-processing parameters, and geometric priors. When arc light, spatter, reflections, and partial occlusion occur simultaneously, these methods are prone to false detections, missed detections, or drift in center-point localization.

To improve the robustness of weld seam perception under severe noise, some studies have begun to introduce learning-driven models for image restoration, denoising, and tracking. Xu et al. [6] applied FT-GAN to restore laser stripe images corrupted by intense arc light and spatter, and integrated this with DCFnet to achieve autonomous weld seam tracking. Their results indicate that integrating image restoration with tracking models can improve robustness in heavy noise welding scenarios. Li and Gao [7] used a stacked denoising autoencoder to perform denoising and feature extraction for GMAW weld profiles under severe noise, demonstrating that deep denoising models can improve weld profile recovery in the presence of noise interference. Although these methods enhance laser stripe recovery under noisy conditions, they usually still depend on additional image restoration, denoising, or tracking procedures, making it difficult to directly output structured keypoint results for multiple types of weld seams.

Driven by advancements in deep learning, the visual perception of weld seams has gradually shifted from rule-based image processing to data-driven feature learning, aiming to improve generalization under complex illumination and across multiple weld seam types. Wang et al. [8] used a semantic segmentation network to extract weld seam regions from laser welding images under severe interference and improved the segmentation accuracy through a lightweight encoder–decoder structure and a channel attention mechanism. Dai et al. [9] proposed WeldNet for precise laser stripe extraction in robotic welding, further demonstrating the value of lightweight deep networks in real-time stripe measurement. Zhao et al. [10] developed a weld seam tracking and detection robot based on an improved YOLOv8s-Seg model. By incorporating a lightweight MobileNetV3 backbone, a C2fGhost structure, and an EMA attention mechanism, their method successfully reduces model complexity while enhancing the capabilities of segmentation-based weld seam perception. Segmentation-based or region-extraction methods can yield relatively complete laser stripes or weld seam regions, but their outputs usually still require centerline fitting, path fitting, or geometric post-processing to produce structured keypoints.

Object detection methods can directly output class labels, confidence scores, and bounding box regression results. YOLO formulates object detection as an end-to-end regression problem, providing a representative one-stage framework for real-time industrial visual inspection [11]. Deng et al. [12] used CenterNet to detect the feature points of three typical weld seam types as object centers, thereby mitigating the negative impact of traditional centerline extraction and explicit geometric rules on detection results. Gao et al. [13] proposed YOLO-Weld, which is based on YOLOv5 and incorporates RepVGG, the NAM attention module, and a lightweight decoupled detection head named RD-Head to improve the speed and stability of weld seam feature point detection under extreme welding noise conditions. Ma et al. [14] proposed Shuffle-YOLO for feature point extraction in complex weld seams, and combined nearest-neighbor matching with cubic B-spline curve fitting to achieve tracking and torch pose adjustment for complex spatially curved weld seams. Zhao et al. [15] proposed the DeepKP framework, which leverages the WeldExt keypoint extraction model and the WeldDenoise denoising model to improve the accuracy of weld seam keypoint extraction under multiple-arc light interferences. Gao et al. [16] further proposed WeldLight, which integrates online data augmentation, a lightweight one-stage network, and an attention module to achieve weld seam classification and feature point localization, while meeting the real-time requirements of weld seam tracking on a CPU platform.

In addition to single-frame detection methods, some studies have sought to improve weld seam perception by incorporating temporal information, multi-source data, and unified keypoint representations. Lu et al. [17] proposed a noise-resistant weld seam tracking algorithm based on a spatiotemporal memory mechanism, leveraging consecutive frame information to enhance the detection stability for narrow weld seams. However, such methods generally depend on temporal sequence continuity. Wei et al. [18] proposed a coarse-to-fine multi-weld detection framework that utilizes RGB images for the coarse localization of weld seam regions and integrates point cloud data for the fine extraction of weld seam edges, thereby improving perception efficiency for workpieces with multiple weld seams. Wang et al. [19] developed a lightweight weld seam keypoint detection and tracking framework based on an improved SimCC, further indicating that unified keypoint representation, precise localization, and real-time inference remain key challenges in welding perception tasks.

Existing studies have made substantial progress in weld seam region segmentation, laser stripe extraction, and feature point detection for typical weld seams. However, when severe noise, multiple weld seam types, small-scale keypoints, and strict center localization requirements coexist, it remains challenging to balance detection accuracy, center localization stability, model complexity, and real-time inference speed [8,9,10,12,13,14,15,16,17,18,19]. Multi-scale feature fusion can enhance the feature representation capability for targets of different scales. FPN builds semantically rich multi-scale feature maps through a top-down pathway and lateral connections, providing an important foundation for small-object detection [20]. SPDConv reduces the loss of fine-grained information in low-resolution images and small-object tasks through space-to-depth rearrangement and non-strided convolution, offering a useful strategy for preserving local structures during early downsampling [21]. NWD models bounding boxes as two-dimensional Gaussian distributions and measures their similarity using the normalized Wasserstein distance, which can alleviate the high sensitivity of tiny object boxes to positional offsets [22]. WIoU assigns regression gradients to samples of different quality through a dynamic focusing mechanism, which helps mitigate the negative impact of low-quality samples on bounding box regression training [23]. Data augmentation methods such as Mosaic, Random Erasing, and CutMix show that multi-image mixing, local erasuring, and region-level mixed perturbations can improve the generalization ability of visual models under complex input conditions [24,25,26].

Based on the above analysis, this paper proposes StripePoint-YOLO, a lightweight weld seam keypoint detection model tailored for severe-noise industrial welding scenarios. The proposed method formulates five typical weld seam types as a unified detection-based keypoint localization task and introduces task-adaptive designs for high-resolution localization, early-stage detail preservation, regression stability, physics-driven noise augmentation, and coarse-scale prediction pruning.

The main contributions of this paper are summarized as follows:

1. A lightweight framework, StripePoint-YOLO, is proposed for unified keypoint detection across five weld seam types. The architecture incorporates a P2 detection head for fine-scale localization and prunes the P5 prediction branch to reduce coarse-scale redundant predictions, while retaining the deep semantic features of P5 for top-down fusion.
2. SPDConv is utilized in the early downsampling stages to preserve local stripe details, facilitating the representation of tiny structural features such as endpoints, intersection points, groove edges, and discontinuity regions.
3. A joint regression loss combining WIoU v3 and NWDLoss is formulated for bounding box regression, effectively mitigating the sensitivity of keypoint bounding boxes to center offsets and low-overlap training samples.
4. Building upon conventional online augmentation, a physics-driven strategy termed OPDDA is specifically designed to simulate real-world welding disturbances like arc light, spatter, and dynamic occlusion. The incorporated keypoint-aware avoidance mechanism preserves the integrity of the supervision signals.
5. Ablation studies, MCE visualizations, and localization error analyses verify the effectiveness of the proposed designs. The final model achieves a practical balance among detection accuracy, localization stability, model size, and inference speed.

The remainder of this paper is organized as follows. Section 2 introduces the experimental platform and dataset. Section 3 describes the proposed StripePoint-YOLO network architecture, key module design, joint loss function, and data augmentation strategy in detail. Section 4 presents the experimental settings, evaluation metrics, ablation studies, result visualization, and localization error analysis, followed by a discussion of detection accuracy, localization error, and inference efficiency. Finally, Section 5 concludes the paper.

## 2. Experimental Platform and Dataset

### 2.1. Experimental System Configuration

**Figure 1:**
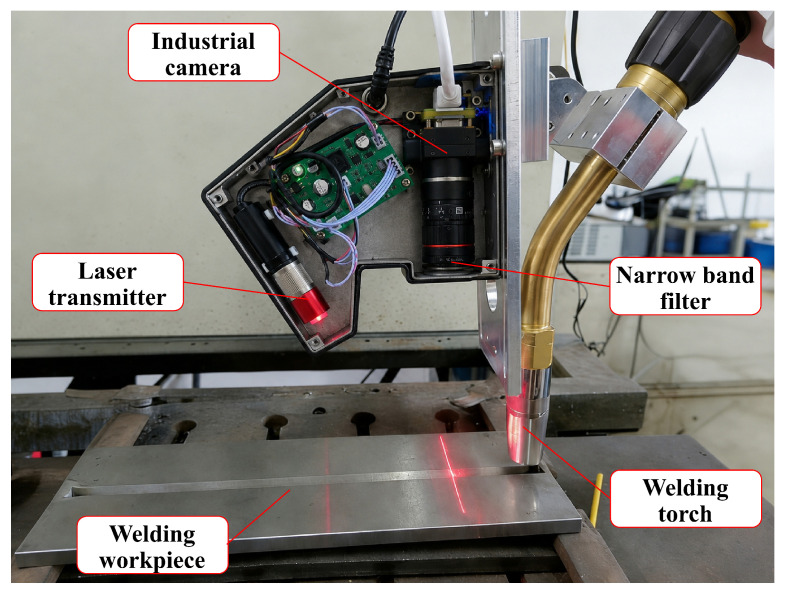
Experimental platform.

To evaluate the keypoint detection performance of StripePoint-YOLO in real-world welding scenarios, a robotic welding vision experimental platform based on single-line structured light was constructed for image acquisition. The platform includes a robotic motion unit, a welding unit, a single-line structured-light vision sensor, and a host computer. The vision sensor is mounted adjacent to the welding torch and maintains a fixed relative pose, enabling the camera to observe both the weld seam area and the adjacent workpiece surface. The experimental platform is shown in Figure 1.

The weld seam images were collected using a custom-developed single-line structured-light vision sensor. The sensor primarily comprises an industrial camera, a single-line laser emitter, a narrow-band optical filter, and a laser intensity adjustment circuit. The industrial camera is a HIKROBOT CB016 board-level camera with a 25 mm focal-length lens. The laser emitter has a central wavelength of approximately 660 nm. A narrow-band filter with the corresponding wavelength range is integrated into the camera lens, which enhances the contrast of the laser stripes and suppresses ambient light interference. The laser intensity can be dynamically adjusted based on the surface reflectivity of the workpiece and the ambient illumination conditions during welding, thereby ensuring the acquisition of relatively stable structured-light images of the weld seam.

During image acquisition, the single-line laser is projected onto the workpiece surface, forming a light stripe across the weld seam region. This stripe characterizes the local geometric profile of the weld seam and its surrounding area. The industrial camera captures raw images from a fixed viewpoint. These raw images capture not only the weld seam profile and laser stripes but also typical industrial interferences such as intense arc light, spatter, and specular reflections. After dataset preparation, all experimental images used in this paper were standardized to a resolution of 1280 × 1080 pixels. During training, the images were resized to 960 × 960 pixels prior to network input. This input size helps retain local structural details of the weld seam while keeping the computational cost acceptable for model training and inference.

StripePoint-YOLO adopts a detection-based keypoint output format. The model does not generate pixel-level segmentation masks or continuous weld seam curves. Instead, it predicts small bounding boxes for key structural locations of the weld seam. Each prediction contains the keypoint class, confidence score, and bounding box coordinates. The center of the predicted bounding box is then extracted as the weld seam keypoint position in the 2D image coordinate system. Through this representation, the model effectively characterizes key structural features, such as weld seam endpoints, intersection points, groove edge points, and local discontinuity regions, thereby providing 2D visual measurements for the subsequent spatial localization of the weld seam.

### 2.2. Weld Seam Dataset and Reconstruction Strategy

In practical industrial welding tasks, the types of weld seams may change frequently, and the number of samples in different classes is often uneven. In this case, the model should not only recognize keypoints from limited training samples, but also maintain a reliable understanding of the weld seam topology. Another problem comes from the image acquisition process of industrial robots. Since the camera records the welding scene continuously, many adjacent frames are highly similar. If these frames are randomly divided into different subsets, similar samples may appear in both the training and validation sets, leading to an overoptimistic evaluation result. To reduce this risk, this paper designs a dataset reconstruction strategy based on hash-based topology preservation and sequence-aware isolation. Perceptual hashing is first used to calculate the Hamming distance between images, and near-duplicate samples are removed according to their visual similarity. When duplicated or highly similar images conflict with each other, the image with more bounding box annotations is retained with priority, because it usually contains more complete keypoint topology information. Then, consecutive frames from the same physical motion trajectory are no longer treated as independent images. Instead, they are regarded as a complete sequence unit and assigned to the same subset during dataset partitioning. In this way, strongly correlated frames are prevented from appearing simultaneously in the training and validation sets. In addition, for pure background images without target keypoints, negative labels are explicitly generated, which helps the model learn background interference and improves its robustness in noisy welding scenes.

After dataset reconstruction, the number of images was reduced from the originally collected 6671 to 6065, including 4852 images for training and 1213 images for validation. The final dataset contains five typical weld seam structures, and all images are unified to a resolution of 1280 × 1080. By removing redundant samples and separating highly correlated image sequences during dataset partitioning, the reconstructed dataset reduces the possibility of overfitting and helps to limit the evaluation bias caused by visually similar adjacent frames. It also provides a more reliable data basis for evaluating weld seam keypoint detection in practical industrial scenarios.

**Figure 2:**
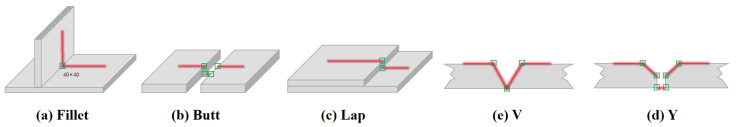
Five weld seam types and keypoint annotations.The green boxes indicate the 40 × 40 pixel bounding boxes used to annotate the weld seam keypoints.

To account for the distinct geometric morphologies and topological relationships exhibited by laser stripes across different weld seams, this paper systematically classifies keypoints according to their local structural characteristics, as detailed in Table 1. A detection-based keypoint annotation strategy is adopted, in which keypoints are labeled using small-scale bounding boxes, and the center of each predicted box is used to represent the keypoint position, as shown in Figure 2. This approach significantly reduces the cost and complexity of manual annotation in industrial scenarios. Simultaneously, it remains compatible with the regression mechanism of advanced object detection algorithms and preserves the representation capability for the local spatial geometry of tiny objects.

## 3. Method

To extract keypoints from weld seam laser stripes without relying on traditional stripe morphology analysis or complex image-continuity constraints, this paper designs a one-stage keypoint detection network based on YOLO11n, tailored for severe welding noise scenarios. In the proposed method, keypoints are annotated using fixed-size bounding boxes, and the center of each object box is used to directly represent the keypoint position. Consequently, the weld seam keypoint localization task is uniformly formulated as a detection-based keypoint modeling problem. To address the requirements for the high-resolution localization of small-scale keypoints, early-stage detail preservation, center regression stability, robustness against physical welding noise, and the suppression of redundant coarse-scale predictions, this paper introduces a P2 detection head, SPDConv, a joint regression loss combining WIoU v3 and NWDLoss, the OPDDA strategy, and a P5 branch pruning strategy that retains deep semantic features, respectively.

### 3.1. YOLO11 Architecture for Weld Seam Keypoint Detection

**Figure 3:**
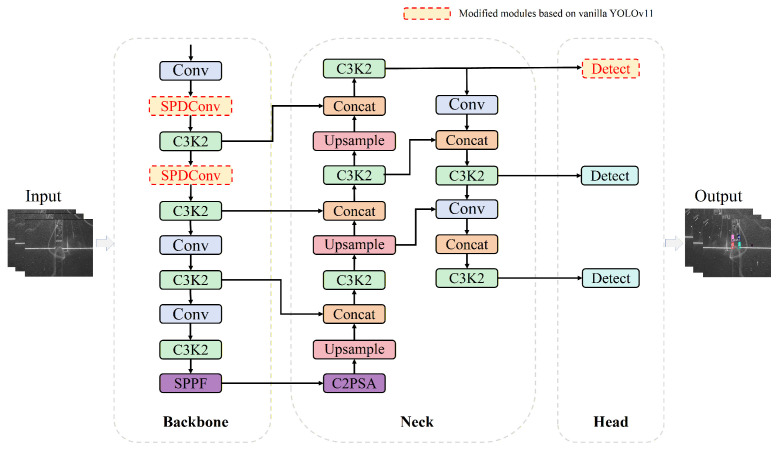
Network architecture of StripePoint-YOLO. The red dashed boxes indicate modules modified from the vanilla YOLOv11. The other colors are used solely to distinguish the module types labeled within the blocks. In the output image, the colored numbered boxes represent the detected keypoint bounding boxes and their corresponding category labels.

Unlike general object detection, the bounding boxes in weld seam keypoint detection are primarily utilized to represent specific point locations. Consequently, even a minute offset in the box center can directly compromise subsequent weld seam geometric measurement and path guidance. The five weld seam types studied in this paper have different numbers of keypoints and different topological relationships. Furthermore, the simultaneous presence of intense arc light, spatter, bright reflective streaks, and partial occlusion in the images renders the valid discriminative structures tiny and highly susceptible to perturbation. Driven by these task characteristics, including small-scale objects, high sensitivity to center offsets, multiple topological categories, and severe noise interference, StripePoint-YOLO incorporates targeted designs in five aspects: high-resolution localization, early-stage detail preservation, regression stability, physics-driven noise augmentation, and coarse-scale branch pruning. The network architecture of StripePoint-YOLO is shown in Figure 3.

The model is built upon YOLO11n. Weld seam keypoints are uniformly represented as fixed small-object boxes, and the center of each predicted box is used as the keypoint coordinate. First, a P2 detection head is introduced so that shallow high-resolution features can directly participate in prediction, improving the distinguishability of tiny structures such as endpoints, intersection points, and local discontinuity regions. Second, SPDConv is adopted in the first two downsampling stages of the backbone. Through space-to-depth rearrangement, SPDConv preserves neighborhood sampling information and alleviates the early-stage detail loss caused by conventional strided convolution [21]. Third, considering that the P5/32 feature map has a relatively low resolution and contributes little to precise center localization when used directly for detection, while also potentially increasing redundant coarse-scale predictions, the P5 detection output layer is removed. The deep semantic features of the P5 layer are retained only for top-down feature fusion. Finally, keypoint prediction is performed at three scales, namely P2, P3, and P4.

During optimization and training, a joint loss based on WIoU v3 and NWDLoss is used to enhance the regression stability of small-scale keypoint bounding boxes under center-offset-sensitive conditions [22,23]. Meanwhile, OPDDA is applied as an online augmentation strategy to simulate common welding disturbances, including arc light, spatter, and dynamic occlusion, thereby improving the model’s adaptability to severe industrial noise [24,25,26].

### 3.2. SPDConv-Based Early Downsampling

The valid discriminative regions for keypoints in line-laser weld seam stripes are extremely small, making them highly susceptible to information loss during the early downsampling stages of the network. In the original YOLO11n, stride-2 convolutions are used for spatial compression during shallow feature extraction. Although this operation can quickly reduce the resolution of feature maps and lower the computational cost of subsequent layers, it may discard some local pixel relationships during sampling, thereby weakening edge continuity and texture integrity around keypoints. Given that the valid discriminative structures in weld seam stripe images are already severely restricted by industrial noise—such as arc light, spatter, and reflections—any further loss of shallow features will drastically degrade the model’s localization stability. To address this issue, SPDConv is introduced into the first two downsampling stages of the backbone to enhance the preservation of fine-grained stripe structures in shallow features.

**Figure 4:**
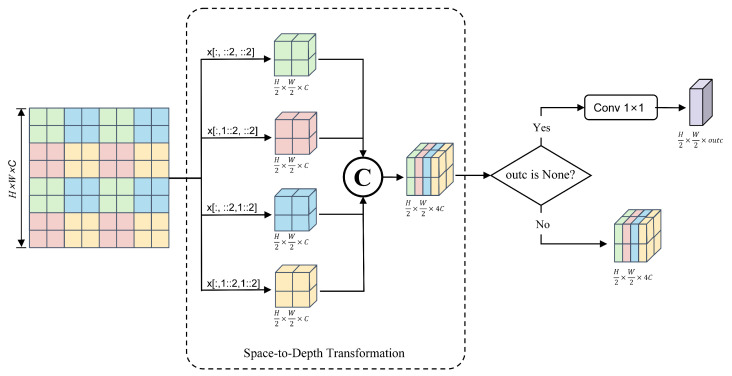
SPDConv module.

SPDConv does not directly perform spatial downsampling through strided convolution. Instead, it first rearranges the input features in the spatial dimension. Let the input feature map be $X\in R^{H\times W\times C}$. The input feature map is divided into four complementary sub-feature maps, as shown in Equation (1). The principle of the SPDConv module is illustrated in Figure 4.

(1) $$\begin{array}{cc} X_{1} & =X(:,0::2,0::2)\\ X_{2} & =X(:,1::2,0::2)\\ X_{3} & =X(:,0::2,1::2)\\ X_{4} & =X(:,1::2,1::2) \end{array}$$

All four sub-feature maps possess a spatial size of $\frac{H}{2}\times \frac{W}{2}$, corresponding to the pixels at the even rows and even columns, odd rows and even columns, even rows and odd columns, and odd rows and odd columns of the original feature map, respectively. These four sub-feature maps are then concatenated along the channel dimension to yield the rearranged feature map $Z\in R^{4C\times \frac{H}{2}\times \frac{W}{2}}$.

(2) $$Z=\mathrm{Concat}(X_{1},X_{2},X_{3},X_{4})$$

While halving the spatial scale, SPDConv maps the local responses originally distributed within each 2 × 2 neighborhood entirely into the channel dimension. After this operation, although the spatial resolution is reduced, the original sampling information within the local neighborhood is still preserved through channel expansion. For the keypoint detection task in this paper, this structure is more effective in retaining subtle differences in bright-line endpoints, intersection edges, and local discontinuity regions.

Following feature rearrangement, a stride-1 convolution is applied for channel fusion and compression. The output feature map is denoted as $Y\in R^{C^{\prime }\times \frac{H}{2}\times \frac{W}{2}}$, as shown in Equation (3), where $C^{\prime }$ represents the number of output channels and $W_{1\times 1}$ denotes a 1 × 1 convolution kernel. If outc is left empty in the module definition, the output of SPDConv is directly set to *Z*, meaning that only the space-to-channel rearrangement is performed without subsequent channel fusion.

(3) $$Y\left(c^{\prime },i,j\right)=\sum_{c=1}^{4C}W_{1\times 1}\left(c^{\prime },c\right)Z\left(c,i,j\right)$$

### 3.3. Joint Regression Loss Optimization

In weld seam keypoint detection, the keypoint position is derived directly from the center of the predicted bounding box; consequently, the quality of bounding box regression directly dictates the accuracy of the keypoint coordinates. Although the task studied in this paper is not strictly a small-object detection problem, its optimization process faces similar challenges, including small effective discriminative regions, high sensitivity to local offsets, and a large number of low-overlap samples. In addition, under interference from intense arc light, spatter, and reflections, the quality of the predicted boxes can fluctuate significantly relative to the ground-truth boxes. Relying solely on conventional IoU-based losses makes it challenging to simultaneously maintain stable constraints during the low-overlap phase and ensure precise localization during the high-overlap phase. Therefore, this paper adopts WIoU v3 from Wise-IoU as the main regression loss and introduces NWD as an auxiliary constraint for low-overlap samples, aiming to improve the regression stability of keypoint bounding boxes under severe industrial noise conditions.

Let the predicted box and the ground-truth box be denoted as $B_{p}$ and $B_{t}$, respectively. Their centers are denoted as $c_{p}$ and $c_{t}$, and their widths and heights are $\left(w_{p},h_{p}\right)$ and $\left(w_{t},h_{t}\right)$, respectively. The standard Intersection over Union (IoU) is defined as follows:

(4) $$\mathrm{IoU}=\frac{|B_{p}\cap B_{t}|}{|B_{p}\cup B_{t}|}$$

(5) $$L_{\mathrm{IoU}}=1-\mathrm{IoU}$$

In Equation (5), $L_{\mathrm{IoU}}$ denotes the basic regression error. By incorporating a center-offset term and a dynamic focusing mechanism, WIoU v3 reweights samples of varying qualities based on the spatial offset between the predicted box and the ground-truth box. Let the width and height of the smallest enclosing box be $c_{w}$ and $c_{h}$, respectively. The center-offset penalty term is then defined in Equation (6). A moving average, ${\mathrm{IoU}}_{\mathrm{mean}}$, is introduced to represent the current training state, and the relative difficulty factor of each sample is defined in Equation (7).

(6) $$D=\exp\left(\frac{∥c_{p}-c_{t}{∥}_{2}^{2}}{c_{w}^{2}+c_{h}^{2}+\varepsilon }\right)$$

(7) $$\beta =\frac{L_{\mathrm{IoU}}}{{\mathrm{IoU}}_{\mathrm{mean}}+\varepsilon }$$

The dynamic gain function and the main regression loss of WIoU v3 are given in Equations (8) and (9), respectively.

(8) $$G\left(\beta \right)=\frac{\beta }{\delta \alpha^{\left(\beta -\delta \right)}+\varepsilon }$$

(9) $$L_{\mathrm{WIoU}}=D\cdot L_{\mathrm{IoU}}\cdot G\left(\beta \right)$$

When the overlap between the predicted box and the ground-truth box is low, relying only on IoU-based losses may lead to unstable supervision. Therefore, NWD is further introduced as an auxiliary constraint. It formulates the discrepancy between boxes as a unified distance that jointly reflects center-position deviation and scale variation, thereby providing smoother gradients during the low-overlap phase. In this task, the geometric distance between the predicted box and the ground-truth box is defined in Equation (10), and the normalized Wasserstein similarity is expressed in Equation (11).

(10) $$d_{w}^{2}={∥c_{p}-c_{t}∥}_{2}^{2}+\frac{{\left(w_{p}-w_{t}\right)}^{2}+{\left(h_{p}-h_{t}\right)}^{2}}{4}$$

(11) $$S_{\mathrm{NWD}}=\exp\left(-\frac{\sqrt{d_{w}^{2}+\varepsilon }}{C}\right)$$

This paper adopts a gated joint optimization strategy combining WIoU v3 and NWD. The joint regression loss is defined in Equation (12), where τ denotes the gating threshold and λ denotes the weight of the auxiliary term.

(12) $$L_{\mathrm{box}}=\left\{\begin{array}{cc} L_{\mathrm{WIoU}}+\lambda \left(1-S_{\mathrm{NWD}}\right),\; & \mathrm{IoU}<\tau \\ L_{\mathrm{WIoU}},\; & \mathrm{IoU}\geq \tau \end{array}\right.$$

### 3.4. Online Data Augmentation

**Figure 5:**
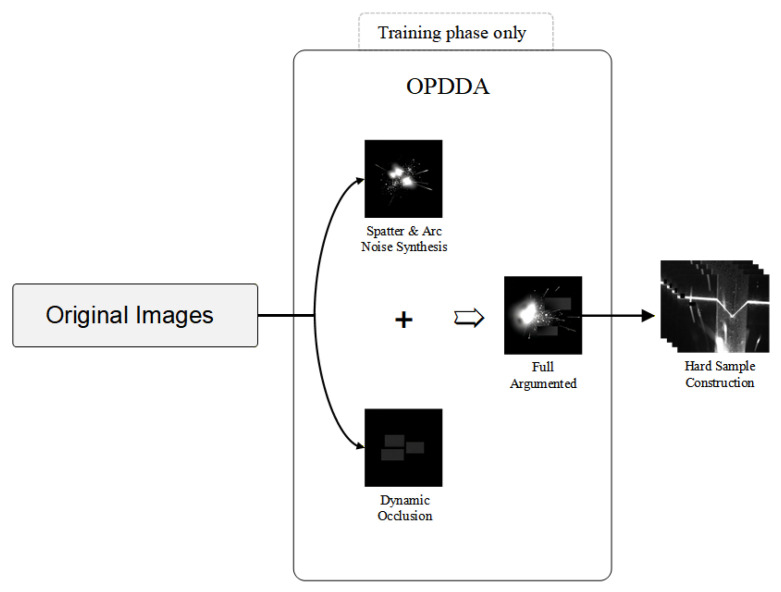
Main workflow of the OPDDA method.

**Figure 6:**
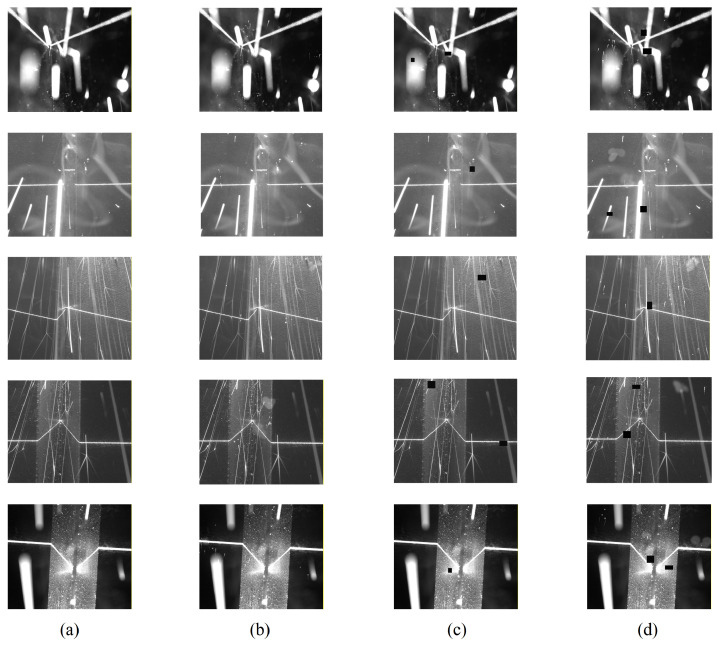
Visual comparison between the original weld seam image and the augmented results produced by different components of OPDDA. (**a**) Original weld seam image. (**b**) Result after adding spatter and arc light noise. (**c**) Result after adding dynamic occlusion. (**d**) Result after simultaneously adding spatter, arc light, and dynamic occlusion.

To improve model training under the condition of limited weld seam samples, it is necessary to augment the weld seam images. First, conventional online augmentation is used to expand the basic geometric and illumination distributions of the samples. Second, an online physics-driven data augmentation method, denoted as OPDDA, is specifically designed for the weld seam keypoint detection task in this paper. This method allows the same original sample to exhibit different forms of physical disturbance across different training iterations. The main workflow of OPDDA is shown in Figure 5. Targeting the typical noise interferences in the welding process, this method focuses on simulating the disruption caused by spatter, arc light, and dynamic occlusion to the laser stripe structures. In this way, the perturbation patterns of the training samples remain consistent with real working conditions, while also providing more representative training samples for the joint regression loss. The visualization results of the proposed OPDDA are shown in Figure 6. The comparison demonstrates that the proposed data augmentation method can preserve the overall structure of the weld seam stripe while simulating typical noise interference in industrial welding scenarios, thereby improving the training efficacy under extreme working conditions.

During training, OPDDA adopts an online random augmentation mechanism. Specifically, the centers of spatter clusters, the positions of arc light regions, and the positions, quantities, sizes, and brightness levels of occlusion blocks are randomly generated during each invocation. As a result, the same image can exhibit different noise distributions across different training epochs. Moreover, dynamic occlusion probabilistically avoids keypoint regions to strike a balance between introducing augmentation perturbations and preserving annotation validity.

## 4. Experimental and Results

This section evaluates the performance of the proposed StripePoint-YOLO for weld seam keypoint detection. The experimental implementation details are first introduced, followed by the evaluation metrics used to assess detection performance, localization accuracy, and inference efficiency. Ablation studies on model components and data augmentation strategies are then conducted to analyze the contribution of the proposed improvements. The proposed method is subsequently compared with several state-of-the-art detection models. Finally, the keypoint localization stability of StripePoint-YOLO under severe welding noise conditions is further analyzed.

### 4.1. Implementation Details

StripePoint-YOLO was implemented in PyTorch 2.10.0 on Windows 11. Model training, inference, and performance evaluation were conducted using an NVIDIA GeForce RTX 5060 Laptop GPU with 8 GB of memory and an Intel Core i7-14650HX CPU. The model parameters are optimized using Stochastic Gradient Descent (SGD), with the learning rate decay trajectory strictly regulated by a cosine annealing scheduler featuring a five-epoch linear warmup. The pretrained weights are initialized from yolo11n.pt.

In industrial welding scenarios, micro-scale weld seam keypoints are highly sensitive to localization accuracy. Therefore, the weights of the bounding box regression term, Distribution Focal Loss (DFL) term, and classification term are set to 12.0, 2.5, and 0.5, respectively. The OPDDA strategy is applied during training with a probability of 0.5. Unlike the common practice of disabling all strong spatial perturbations during the late-stage fine-tuning phase, this paper adopts a decoupled augmentation mechanism for standard augmentation and physics-driven augmentation. Standard Mosaic augmentation is disabled in the last 20 epochs to mitigate data distribution shifts during the final training phase. In contrast, the proposed online data augmentation strategy is retained throughout the entire training process to reduce overfitting to local pixel patterns. Some of the training parameters of StripePoint-YOLO are listed in Table 2. These configuration settings are essential for reproducing the experiments and achieving comparable performance.

### 4.2. Evaluation Metrics

This paper evaluates StripePoint-YOLO in terms of three aspects: detection performance, localization accuracy, and inference efficiency. Detection performance is characterized by mAP@50-95; localization accuracy is evaluated using the Mean Center Error (MCE); and inference efficiency is measured using the number of parameters (Params), GFLOPs, Latency, and FPS.

For detection performance, mAP@50-95 is adopted to evaluate the overall detection quality of the model under different IoU thresholds. Compared to mAP@50, mAP@50-95 is more sensitive to localization errors and can more effectively reflect the overall detection quality of the model under multi-threshold constraints in our specific detection task. Given that mAP@50 has already reached a saturated high level in our experiments, its discriminative power in evaluating model superiority is limited. Therefore, it is no longer used as the primary evaluation metric.

**Figure 7:**
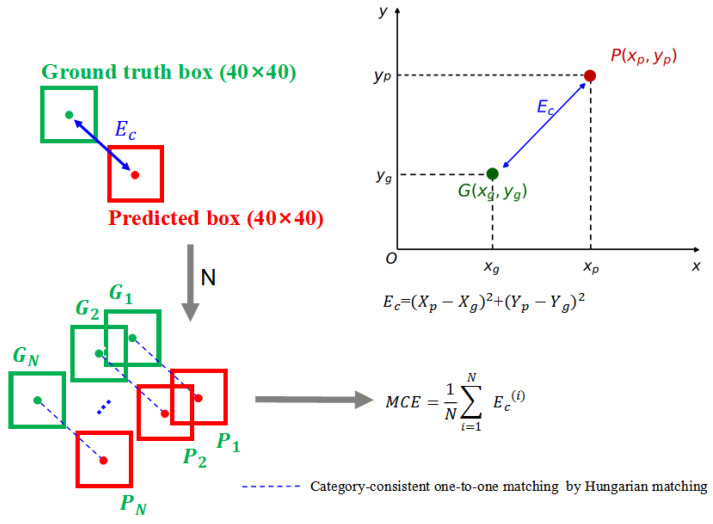
Principle of MCE calculation.

For localization accuracy, MCE is used to calculate the average Euclidean distance between the center of the predicted box and the center of the ground-truth box. Let the predicted box center be $\left(x_{p},y_{p}\right)$ and the ground-truth box center be $\left(x_{g},y_{g}\right)$. The center error of a single keypoint is defined in Equation (13), and the Mean Center Error of all valid matched keypoints is defined in Equation (14).

(13) $$E_{c}=\sqrt{{\left(x_{p}-x_{g}\right)}^{2}+{\left(y_{p}-y_{g}\right)}^{2}}$$

(14) $$\mathrm{MCE}=\frac{1}{N}\sum_{i=1}^{N}E_{c}^{\left(i\right)}$$

where *N* denotes the number of valid matched keypoints included in the calculation. Considering that keypoints of the same class may be densely distributed within a local region, directly employing greedy matching can easily lead to mismatches caused by locally optimal assignments. To mitigate the impact of this issue on the center error statistics, this paper constructs a cost matrix based on the Euclidean distance between predicted box centers and ground-truth box centers under the constraint of class consistency. The Hungarian algorithm is then used to establish a one-to-one correspondence between the predicted results and the ground-truth annotations, based on which MCE is calculated, as shown in Figure 7. A smaller MCE value indicates a smaller keypoint center localization error.

To further evaluate the tail-end localization stability of the model on difficult samples, this paper introduces the 95th-percentile Mean Center Error P95-MCE. Let the set of center errors for all valid matched keypoints be $E=\{e_{i}|i=1,2,\ldots ,N\}$. After sorting *E* in ascending order, P95-MCE is defined as the 95th percentile of this error set: $P95-\mathrm{MCE}={\mathrm{Percentile}}_{95}\left(E\right)$.

Compared to localization accuracy based on a fixed pixel threshold, P95-MCE eliminates the reliance on a manually predefined tolerance range, thereby providing a more direct reflection of the upper error bound for 95% of the keypoint samples. A smaller value indicates better localization stability in difficult scenarios, such as intense arc light, spatter, reflections, and densely distributed keypoint regions.

Collectively, these metrics enable a comprehensive evaluation of the model’s detection quality, average localization accuracy, tail-error control capability, and deployment efficiency in real-world severe-noise industrial scenarios.

### 4.3. Ablation Study

To evaluate the specific contribution of each component in StripePoint-YOLO to weld seam keypoint detection, ablation studies are conducted, with the results summarized in Table 3. The original YOLO11n is used as the baseline model. For a fair comparison, all experiments follow the same data split, input resolution, and number of training epochs. Under these unified settings, the ablation study mainly evaluates the effects of the P2 high-resolution detection head, the joint regression loss combining WIoU v3 and NWDLoss, the early-stage detail preservation enabled by SPDConv, and the P5 branch pruning strategy that retains deep semantic features for top-down feature fusion.

As shown in Table 3, after introducing P2, mAP@50-95 increases from 73.09% to 74.16%, indicating that the high-resolution branch can provide richer spatial details for small-scale keypoints, although the number of parameters and GFLOPs also increase. Applying WIoU v3 and NWDLoss independently yields an mAP@50-95 of 79.08% and a Mean MCE of 2.29 px, demonstrating that optimizing regression stability is particularly crucial for keypoint bounding boxes sensitive to center offsets. With the combined effect of P2 and the joint loss, Model H achieves the highest mAP@50-95 of 80.63%, but its parameter count and computational cost remain relatively high. After SPDConv is introduced, Model I maintains an mAP@50-95 above 80% while reducing GFLOPs from 23.98 to 21.05, indicating that the early-stage detail preservation design can reduce information loss during downsampling and improve computational efficiency. The final Model M achieves an mAP@50-95 of 80.46% and a Mean MCE of 2.33 px after pruning the P5 detection branch, with only 1.86 M parameters and 19.42 GFLOPs. Compared with Model H, Model M shows only a 0.17 percentage-point decrease in mAP@50-95, while reducing the number of parameters by 36.1% and GFLOPs by 19.0%. This suggests that the P5 detection output contributes little to the localization of tiny weld seam keypoints in this task. Retaining the semantic features of P5 while removing its prediction branch produces a more favorable balance between detection accuracy, model complexity, and image-level inference efficiency.

To further verify the impact of the OPDDA data augmentation strategy on model performance, an ablation study on the dynamic occlusion avoidance probability is conducted while keeping the network architecture fixed. In the experiment, spatter and arc light augmentation are kept unchanged, while only the probability that dynamic occlusion is forced to avoid keypoint regions is varied. The probabilities are set to 100%, 90%, 80%, 75%, and 60%, respectively. The experimental results are shown in Table 4.

As shown in Table 4, a higher avoidance probability for dynamic occlusion does not necessarily lead to better performance. If the avoidance probability is too low, the effective supervision information around keypoints may be damaged. If it is too high, the strength of occlusion perturbation is weakened, making the augmented samples insufficiently challenging. An avoidance probability of 80% achieves a better balance between noise perturbation and keypoint information preservation.

**Figure 8:**
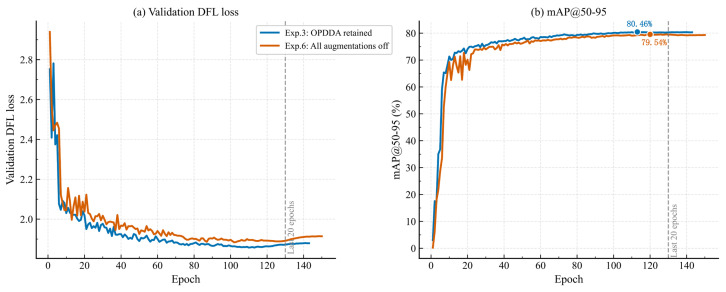
Comparison results of different late-stage training augmentation scheduling strategies. (**a**) Validation DFL loss. (**b**) mAP@50-95. Exp. 3 indicates that standard Mosaic augmentation is disabled during the last 20 epochs while OPDDA is continuously retained. Exp. 6 indicates that all data augmentation is disabled during the last 20 epochs. The results show that continuously retaining OPDDA leads to lower validation loss and higher detection accuracy.

To further analyze the effect of the late-stage augmentation scheduling strategy, this paper compares two training settings under the 80% avoidance probability: continuously retaining OPDDA and disabling all data augmentation in the last 20 epochs. The comparison results are shown in Figure 8. OPDDA differs from standard Mosaic augmentation in its working mechanism. Standard Mosaic mainly changes the global image distribution, whereas the proposed data augmentation strategy serves as an indispensable adversarial regularization mechanism. It suppresses feature inertia in tiny objects and forces the network to maintain its ability to reason about the global geometric topology.

### 4.4. Comparative Experiments

To compare StripePoint-YOLO with different detection architectures, YOLO12n, YOLOv10n, YOLO11n, and RT-DETR-L were selected as reference models. The YOLO models are lightweight one-stage detectors, whereas RT-DETR-L adopts a transformer-based end-to-end architecture with query-based prediction and Hungarian matching.

The shared training and evaluation settings were kept consistent across the comparison models, while architecture-specific optimization mechanisms were retained. Due to the higher memory requirement of RT-DETR-L, its physical batch size was set to 1, whereas the YOLO models used a batch size of 8; the nominal batch size was kept consistent across all models. RT-DETR-L also retained its native matching, loss calculation, and end-to-end prediction strategy.

As shown in Table 5, the YOLO baselines provide relatively high inference speeds but lower detection and center localization performance. RT-DETR-L achieves relatively competitive accuracy, but its model size, computational cost, and inference latency are considerably higher. Overall, StripePoint-YOLO provides a more favorable balance among detection accuracy, localization performance, model complexity, and inference efficiency.

### 4.5. Result Visualization and Localization Error Analysis

**Figure 9:**
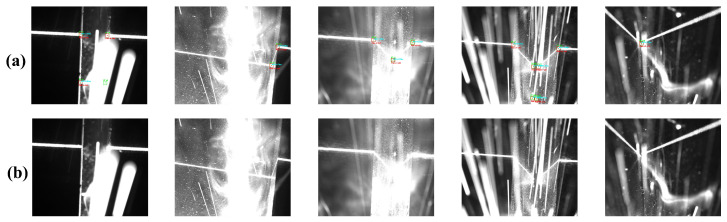
MCE visualization results of difficult samples from different weld seam types: (**a**) Keypoint detection and MCE visualization results for five weld seam types. (**b**) Corresponding original images. Green boxes indicate ground-truth annotations, red boxes indicate predictions, and the values in the upper-left corner of each image represent the center error of matched keypoints.

Figure 9 presents the MCE visualization results of difficult samples from different weld seam types, further verifying the localization performance of the proposed StripePoint-YOLO model in complex welding scenarios. Figure 9a shows the model prediction results together with center error visualization, while Figure 9b shows the corresponding original weld seam images. From a qualitative perspective, the model can adapt well to complex interference, such as intense arc light, spatter, overexposed reflections, and vertical bright streaks. In most cases, it can still accurately identify weld seam keypoint positions while maintaining relatively small center localization errors. Although certain spatters and reflective streaks exhibit high local morphological similarities to laser stripes, the model successfully distinguishes valid keypoint regions from complex backgrounds, demonstrating robust anti-interference capabilities.

As shown in Figure 9, the detection difficulty varies among different weld seam types. For butt welds, several keypoints are located at edge intersections. When these local regions are affected by strong reflection or spatter occlusion, individual keypoints may be missed, and redundant predictions may also appear. Lap welds have fewer keypoints, generally located where the lap edge intersects with the laser stripe. When the edge contour is disturbed by overexposure or sharp dark–bright transitions, detection in these local regions may become unstable. For V-shaped welds, the relatively clear visual features of the left and right endpoints allow the model to localize them stably. The central intersection point remains more challenging to detect. It lies in the convergence region of the groove and is highly susceptible to arc light and reflective textures, which may lead to center offsets or missed detections. Y-shaped welds exhibit the most complex structures. Their keypoints are densely distributed, especially in the middle and lower regions. Exacerbated by severe noise, reflective streaks, and narrow keypoint spacing, these regions are highly prone to redundant predictions or elevated local MCE values. Fillet welds contain only one keypoint, showing no obvious missed or false detections in the sampled visualization results. The structural complexity of the weld seam and the spatial density of keypoints both have a significant impact on overall detection stability.

The MCE visualization in Figure 9a further shows that, for most matched keypoints, the centers of the predicted boxes remain close to the centers of the ground-truth boxes. This indicates that StripePoint-YOLO not only detects keypoint classes effectively, but also provides good center localization. In regions with mild noise or clearer structures, the model generally achieves smaller MCE values. However, under severe overexposure, dense spatter, bright streaks crossing keypoint neighborhoods, or closely spaced keypoints, local MCE tends to increase. This suggests that complex optical interference in welding images weakens the texture and geometric information in the keypoint neighborhood, affecting the model’s ability to precisely determine keypoint centers.

Notably, since MCE primarily measures the center distance between successfully matched keypoints, it cannot serve as a standalone metric for evaluating missed or redundant predictions. For example, when GT_num is greater than Matched_num in an image, Mean MCE only calculates the localization error of the matched keypoints, while unmatched missed keypoints are not directly included in the calculation. Similarly, when Pred_num is greater than GT_num, extra predictions are not directly reflected in Mean MCE.

**Figure 10:**
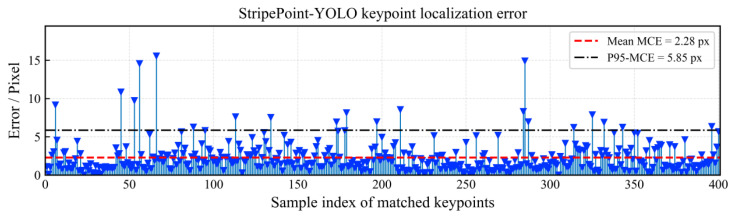
Keypoint localization error distribution of StripePoint-YOLO.

As shown in Figure 10, the localization errors of StripePoint-YOLO on matched keypoints are highly concentrated. Specifically, the Mean Center Error (MCE) for the 400 sampled keypoints is 2.28 px, with the vast majority of samples falling within a low-error interval. This indicates that the model can maintain stable pixel-level localization in complex weld seam images. The P95-MCE of the sampled results is 5.85 px, suggesting that the center offsets of the vast majority of keypoints are controlled within a small range and that the model achieves good localization reliability under severe noise conditions. Nevertheless, a small number of error peaks are observed in the figure, with the errors of a few samples exceeding 10 px. These significant deviations primarily stem from difficult scenarios such as arc-induced overexposure, spatter occlusion, intense local reflections, and blurred boundaries in the keypoint neighborhood. Overall, the error distribution is dominated by low-error samples, with large offsets occurring only in a few severely perturbed samples, verifying the stable localization capability of StripePoint-YOLO for keypoints across multiple weld seam types.

Figure 10 visualizes the distribution of keypoint localization errors, with the 400 matched keypoints sampled from the valid matching results of the validation set. The Mean MCE and P95-MCE in this figure primarily describe the error distribution of these specific samples. The statistics in Table 6 rely on the complete validation set rather than the visualization subset. Comprising 3981 ground-truth keypoints and 3958 successfully matched keypoints, this full validation set provides the Median MCE, P90-MCE, and P95-MCE reported in Table 6 as the final localization error evaluation metrics. The slight discrepancies in error values between Figure 10 and Table 5 stem naturally from their different statistical scopes.

As shown in Table 6, StripePoint-YOLO achieves high keypoint matching rates across different weld seam types, reaching an overall rate of 99.42%. The model consistently detects key structural points in multiple weld seam forms. Butt, V-shaped, Y-shaped, and fillet welds all exceed a 99% matching rate, reflecting strong adaptability to various topological structures. The matching rate for lap welds is slightly lower at 97.37%. Missed detections in this category likely result from the proximity of the weld boundary to the workpiece edge, intense local reflections, and weak texture information in the keypoint neighborhood.

Regarding localization error distribution, the model achieves a Median MCE of 1.72 px, a P90-MCE of 4.29 px, and a P95-MCE of 5.72 px across the entire dataset, indicating tightly constrained center errors for most keypoints. V-shaped welds exhibit the lowest localization errors, with a Median MCE of 1.40 px and a P95-MCE of 3.94 px. The distinct geometric features of this weld type enable highly stable center localization. The P95-MCE values for Y-shaped and fillet welds are 6.12 px and 6.27 px, respectively, surpassing other types. Complex groove structures, local overexposure, spatter occlusion, and blurred boundaries in the keypoint neighborhood contribute to these elevated tail errors. StripePoint-YOLO maintains excellent detection completeness and localization stability across all five weld seam types, with the few large errors primarily confined to difficult samples featuring complex topological structures or severe visual interference.

## 5. Future Work

Building on the current image-plane keypoint localization results, future work will extend the evaluation to a wider range of imaging and workpiece conditions. Controlled image-acquisition experiments will be conducted to examine how surface characteristics, specimen geometry, laser stripe quality, camera configuration, and laser wavelength affect stripe visibility and localization stability. This will also support the detection of plate edges and deformation-related features and provide a basis for assessing the transferability of the model across different structured-light sensing systems.

Camera and laser-plane calibration will subsequently be introduced to reconstruct the detected keypoints in metric three-dimensional coordinates and transform them into the robot coordinate system. The reconstructed weld seam geometry will be used for robotic path correction and controlled welding tests covering different materials, joint geometries, and process conditions. By comparing image-plane localization errors with physical path deviations and post-weld inspection results, the influence of keypoint detection accuracy on path-following performance, weld defects, and heat-affected-zone consistency can be further evaluated.

## 6. Conclusions

To address the instability of weld seam keypoint detection caused by intense arc light, spatter, reflections, and partial occlusion in complex industrial welding scenarios, this paper proposes a lightweight detection model named StripePoint-YOLO. The proposed method formulates five typical weld seam types as a unified detection-based keypoint localization task, and incorporates structural designs focusing on high-resolution representation of small-scale keypoints, early-stage detail preservation, stable center regression, adaptation to physical noise, and suppression of redundant coarse-scale predictions. Specifically, the P2 detection head is introduced to enhance the ability of shallow features to represent tiny endpoints, intersection points, and edge points; SPDConv is adopted to mitigate the loss of local stripe information during early-stage downsampling; a joint regression loss combining WIoU v3 and NWDLoss is utilized to improve the regression stability of keypoint bounding boxes under low-overlap and severe-interference conditions; and the P5 detection branch is pruned while retaining its semantic fusion to minimize the redundant influence of the coarse-scale detection branch on fine center localization.

Experimental results demonstrate that StripePoint-YOLO achieves an mAP@50-95 of 80.46% and a Mean MCE of 2.33 px on the five-class weld seam keypoint dataset constructed in this study. The model features only 1.86 M parameters and 19.42 GFLOPs, and achieves an inference speed of 159.80 FPS on the reported hardware configuration. Further MCE visualization and localization error statistics on the entire validation set reveal an overall keypoint matching rate of 99.42%, alongside Median MCE, P90-MCE, and P95-MCE values of 1.72 px, 4.29 px, and 5.72 px, respectively. These comprehensive evaluations indicate that StripePoint-YOLO can maintain high keypoint detection completeness and pixel-level center localization stability across multiple weld seam types and complex noise conditions. The proposed method provides an accurate and efficient approach for weld seam keypoint detection, offering a practical visual basis for subsequent weld seam geometric measurement and robotic welding perception research.

## Figures and Tables

**Table 1:** Division of the weld seam dataset.

Dataset Split	Fillet	Butt	Lap	V	Y	Total
Train set	1024	964	990	981	893	4852
Val set	229	230	230	257	267	1213
Total	1253	1194	1220	1238	1160	6065

**Table 2:** Parameter settings of StripePoint-YOLO training.

Parameters	Values	Parameters	Values
Operating System	Windows 11	PyTorch	2.10.0
CUDA	12.8	Python	3.11.14
Ultralytics	8.3.163	opencv-python	4.13.0.90
Epochs	150	Optimizer	SGD
Initial learning rate	0.01	Image size	960×960 px
Batch size	8	Validation IoU threshold	0.7
Box loss gain	12.0	DFL loss gain	2.5
Weight decay	0.0005	Warmup momentum	0.8
Warmup epochs	5	Warmup bias learning rate	0.1

**Table 3:** Ablation experiment results.

Model	P2	WIoU + NWDLoss	WIoU	NWDLoss	SPDConv	NoP5	mAP@50-95 (%)	Mean MCE (px)	Params (M)	GFLOPs	Latency (ms)	FPS
Baseline (YOLO11n)							73.09	2.85	2.59	14.24	4.360±0.030	229.36
Model A	✓						74.16	2.30	2.91	23.98	7.417±0.341	135.08
Model B		✓					79.08	2.29	2.59	14.24	4.375±0.101	228.67
Model C			✓				79.57	2.33	2.59	14.24	4.495±0.152	222.46
Model D				✓			77.75	2.33	2.59	14.24	4.403±0.089	227.12
Model E	✓		✓				79.61	2.26	2.90	23.98	6.960±0.034	143.68
Model F	✓		✓		✓		80.61	2.38	2.76	21.05	6.728±0.122	148.64
Model G	✓		✓		✓	✓	80.34	2.39	1.86	19.42	6.212±0.047	160.97
Model H	✓	✓					80.63	2.40	2.91	23.98	7.854±1.285	129.63
Model I	✓	✓			✓		80.21	2.36	2.76	21.05	6.848±0.133	146.08
Model M (Ours)	✓	✓			✓	✓	80.46	2.33	1.86	19.42	6.258±0.077	159.80

**Table 4:** Ablation experiment results of the OPDDA data augmentation strategy.

Experiment	Occlusion Avoidance Probability	Augmentation in the Last 20 Epochs	Final mAP@50-95	Best mAP@50-95	Final val/dfl_loss	Minimum val/dfl_loss
Exp. 1	100%	OPDDA retained	79.871%	79.967%	1.90182	1.87291
Exp. 2	90%	OPDDA retained	80.041%	80.145%	1.90328	1.87403
Exp. 3	80%	OPDDA retained	**80.32%**	**80.46%**	**1.87922**	**1.85716**
Exp. 4	60%	OPDDA retained	78.789%	79.303%	1.96814	1.90746
Exp. 5	75%	OPDDA retained	78.636%	79.525%	1.98383	1.90198
Exp. 6	80%	No Enhancements	79.318%	79.543%	1.91402	1.88374

Note: Bold values indicate the best result in each column. Best mAP@50-95 refers to the highest validation mAP@50-95 achieved during training, while Final mAP@50-95 refers to the validation result obtained in the final epoch. Exp. 6 indicates that, under an 80% probability of keypoint occlusion avoidance, all data augmentation was disabled during the last 20 epochs, consistent with the epoch setting used for disabling standard Mosaic augmentation during training.

**Table 5:** Comparison results with mainstream object detection models.

Model	mAP@50-95 (%)	Mean MCE (px)	Params (M)	GFLOPs	Latency (ms)	FPS
YOLO12n	71.77	2.58	2.56	14.26	8.794±0.052	113.71
YOLOv10n	73.77	2.69	2.27	14.72	4.620±0.035	216.44
YOLO11n (baseline)	73.09	2.85	2.59	14.24	4.360±0.030	229.36
RT-DETR-L	74.14	2.94	32.02	228.58	40.113±1.458	24.93
StripePoint-YOLO (Ours)	80.46	2.33	1.86	19.42	6.258±0.077	159.80

**Table 6:** Keypoint localization error statistics of StripePoint-YOLO for different weld seam types.

Weld Type	Keypoint Classes	GT Keypoints	Matched Keypoints	Match Rate (%)	Median MCE (px)	P90-MCE (px)	P95-MCE (px)
Fillet	c15	1602	1598	99.7503	1.8895	4.8041	6.2748
Butt	c3, c4, c5, c6	920	912	99.1304	1.652	3.8307	5.0153
Lap	c13, c14	228	222	97.3684	1.6738	3.6267	4.2649
V	c0, c1, c2	460	458	99.5652	1.4005	3.1262	3.9436
Y	c7, c8, c9, c10, c11, c12	771	768	99.6109	1.6947	4.7325	6.1186
Overall	all	3981	3958	99.42	1.72	4.29	5.72

## Data Availability

The raw data supporting the conclusions of this article will be made available by the authors on request.
